# GALAD Score for the Diagnosis of Hepatocellular Carcinoma in Sub-Saharan Africa: A Validation Study in Ghanaian Patients

**DOI:** 10.1158/2767-9764.CRC-24-0227

**Published:** 2024-10-10

**Authors:** Yvonne Ayerki Nartey, Ju Dong Yang, Tyler J. Zemla, Joshua Ayawin, Shadrack Osei Asibey, Mohamed El-Kassas, Sally Afua Bampoh, Amoako Duah, Adwoa Agyei-Nkansah, Yaw Asante Awuku, Mary Yeboah Afihene, Hiroyuki Yamada, Jun Yin, Amelie Plymoth, Lewis R. Roberts

**Affiliations:** 1 Department of Internal Medicine, Cape Coast Teaching Hospital, Cape Coast, Ghana.; 2 Department of Internal Medicine and Therapeutics, School of Medical Sciences, University of Cape Coast, Cape Coast, Ghana.; 3 Karsh Division of Gastroenterology and Hepatology, Cedars-Sinai Medical Center, Los Angeles, California.; 4 Alliance Statistics and Data Management Center, Mayo Clinic, Rochester, Minnesota.; 5 Department of Internal Medicine, Komfo-Anokye Teaching Hospital, Kumasi, Ghana.; 6 Department of Health Statistics, Kumasi Technical University, Kumasi, Ghana.; 7 Department of Endemic Medicine, Faculty of Medicine, Helwan University, Cairo, Egypt.; 8 Department of Internal Medicine, Greater Accra Regional Hospital, Accra, Ghana.; 9 Department of Internal Medicine, University of Ghana Medical Center, Accra, Ghana.; 10 Department of Internal Medicine, University of Ghana Medical School, Accra, Ghana.; 11 Department of Internal Medicine, University of Health and Allied Sciences, Ho, Ghana.; 12 Department of Internal Medicine, Kwame Nkrumah University of Science and Technology, Kumasi, Ghana.; 13 Division of In Vitro Diagnostics, FUJIFILM Corporation, Tokyo, Japan.; 14 Department of Biostatistics and Bioinformatics, Moffitt Cancer Center, Tampa, Florida.; 15 European Centre for Disease Prevention and Control (ECDC), Stockholm, Sweden.; 16 Division of Gastroenterology and Hepatology, Mayo Clinic, Rochester, Minnesota.

## Abstract

Hepatocellular carcinoma (HCC) is one of the leading causes of cancer-related mortality worldwide including sub-Saharan Africa. The GALAD score, derived from **G**ender, **A**ge, ***L****ens culinaris* agglutinin-reactive fraction of alpha fetoprotein, **A**lpha fetoprotein, and **D**es-carboxy-prothrombin, has high accuracy in diagnosing HCC in Asia, Europe, and North America; however, it has not been validated in an African cohort. The aim of this study was to assess the performance of the GALAD score in the diagnosis of HCC in sub-Saharan Africa. Clinical data from patients with cirrhosis (*n* = 93) or HCC (*n* = 78) from outpatient hepatology clinics at three teaching hospitals in Ghana were abstracted, and serum samples were analyzed. A logistic regression model predicting HCC status based on the GALAD score was constructed to obtain the ROC curve for GALAD. The AUC with 95% confidence interval (CI) was calculated. The median GALAD score was higher among patients with HCC versus cirrhosis controls (8.0 vs. −4.1, *P* < 0.01). The AUC of the GALAD score for HCC detection was 0.86 (95% CI, 0.79–0.92). At a cut-off value of −0.37, the GALAD score had a sensitivity of 0.81 and a specificity of 0.86. The AUC (95% CI) was 0.87 (0.80–0.95) and 0.81 (0.67–0.94) in hepatitis B virus–positive and hepatitis B virus–negative patients, respectively. The GALAD score has a high accuracy for HCC detection. It has great potential to improve HCC surveillance in sub-Saharan Africa where imaging resources are limited.

**Significance:**

The GALAD score or its relevant modifications have the potential to aid in improving HCC surveillance efforts in low-resource settings in sub-Saharan Africa. This could enhance early detection rates of HCC and potentially improve survival rates in resource-limited settings.

## Introduction

Hepatocellular carcinoma (HCC) is one of the leading causes of cancer-related death worldwide (1). In 2020, there were 905,700 cases of HCC diagnosed globally and 830,200 deaths (2). The burden of HCC is unequal worldwide, as evidenced by an age-standardized incidence rate of 11.9 per 100,000 in Southeast Asia, 9.0 per 100,000 in Northern Africa, and 6.3 per 100,000 in sub-Saharan Africa. In Africa, HCC is the second most common cancer in men and the sixth in women (3). HCC occurs at a younger age among Africans, with a median age of diagnosis of 46 years (4, 5). Hepatitis C virus (HCV) is the predominant risk factor for HCC in Northern Africa, whereas hepatitis B virus (HBV) is the most common cause in other African regions (6). The median survival of patients with HCC in sub-Saharan African countries including Nigeria, Ghana, Ivory Coast, and Cameroon is 2.5 months, primarily due to the advanced stage of disease at presentation and limited therapeutic options in this setting (5).

HCC surveillance of at-risk patients has been shown to improve recognition of early-stage disease and patient survival (7). A six-monthly abdominal ultrasound, in combination with alpha fetoprotein (AFP) measurement, is recommended as a screening method for at-risk groups or target populations, including those with liver cirrhosis, by the European Association for the Study of the Liver and the American Association for the Study of Liver Diseases (8, 9). The adequacy of ultrasound in HCC screening depends on factors such as body mass index, underlying etiology, and operator skill (10). Tumor biomarkers, such as AFP and des-gamma-carboxy prothrombin (DCP), are also used in clinical practice to screen and diagnose HCC. AFP in combination with ultrasound shows improved sensitivity in the diagnosis of HCC, compared with using ultrasound alone [65% sensitivity vs. 45% sensitivity (11, 12)]. However, AFP levels are influenced by several factors including the etiology and type of HCC, and levels may be elevated in patients with active hepatitis in the absence of liver cancer (13, 14).

To improve accuracy in the detection of HCC, the GALAD model, a triple biomarker model that combines gender, age, and the levels of three serologic biomarkers: AFP, *Lens culinaris* agglutinin-reactive fraction of AFP (AFP-L3%), and DCP, has been studied and subsequently validated in Europe, Asia, and the United States of America (15–19). The performance of the GALAD score for HCC has been found to vary by etiology, race, and stage of HCC. For example, a higher AUC is seen in HCC cases associated with HCV and non-viral etiology (20, 21), compared with HBV. Furthermore, the sensitivity, specificity, and AUC of the GALAD score are lower for early stage compared with any-stage HCC when using the Barcelona Clinic Liver Cancer (BCLC) staging (20). Additionally, sensitivity and specificity of the score were variable between patients from Asia, America, and Europe (19, 20). The performance of the GALAD score is yet to be assessed in sub-Saharan Africa, where the underlying etiology of HCC is predominantly HBV. This study aimed to examine the accuracy of the GALAD serologic model in the diagnosis of HCC in a Ghanaian cohort.

## Materials and Methods

### Study cohort

One hundred and seventy-one study participants were enrolled from outpatient hepatology clinics at the Cape Coast Teaching Hospital, Korle Bu Teaching Hospital, and Komfo-Anokye Teaching Hospital in Ghana between January 2017 and December 2018. Sociodemographic and clinical data were abstracted from patient medical records. A total of 171 patients were enrolled for this study. Sample size calculation for diagnostic accuracy studies with 95% confidence interval (CI) and expected sensitivity and specificity of 80% (22) yielded a minimum sample size requirement of 123 study participants in total. The control group consisted of patients with cirrhosis (*n* = 93). Patients with cirrhosis were selected at the control group due to their increased risk of HCC. Inclusion criteria for controls were (i) adults ages 18 years or above; (ii) a diagnosis of cirrhosis made by a gastroenterologist/hepatologist based on clinical history, physical examination, laboratory, and ultrasound findings. Laboratory determinants for cirrhosis included noninvasive tools such as an aspartate aminotransferase–platelet ratio index score of >2 or Fibrosis-4 index of >3.25. Ultrasound findings for cirrhosis included an irregular or nodular hepatic surface, echogenic liver, the presence of portal collaterals, and other morphologic features of cirrhosis. Any patient with a focal hepatic mass in addition to features of cirrhosis was investigated for HCC. The case group consisted of patients with HCC (*n* = 78). The inclusion criteria for cases were (i) adults ages 18 years or above; (ii) a diagnosis of HCC made by a gastroenterologist/hepatologist based on abdominal imaging. Exclusion criterion was (i) failure to provide informed consent. Furthermore, for cases and controls, additional clinical history and investigations to determine the underlying cause of cirrhosis and HCC were obtained. The presence of HBsAg (HBV), anti-HCV ± HCV RNA (HCV), significant alcohol use history of >20 g per day (alcohol-associated liver disease), and the presence of metabolic syndrome with alcohol consumption of less than 20 g per day (metabolic dysfunction–associated steatotic liver disease or MASLD) were considered potential etiologies. Testing of serum samples for AFP, AFP-L3%, and DCP was conducted using a microchip capillary electrophoresis and liquid-phase binding assay by FUJIFILM Wako Chemicals Europe GmbH.

### Statistical analysis

Patient baseline characteristics were compared between case group subjects with HCC and control group subjects with cirrhosis. Continuous variables were presented as medians with interquartile percentiles, whereas categorical variables were expressed as counts with percentages. Univariate comparisons were performed using Wilcoxon rank-sum tests for continuous variables and Pearson χ^2^ tests for categorical variables.

The following previously published equation was used for the GALAD model: *Z* = −10.08 + 0.09 × age + 1.67 × sex + 2.34 log_10_ (AFP) + 0.04 × AFP − L3 + 1.33 × log_10_ (DCP), in which sex = 1 for males and 0 for females (15). A logistic regression model predicting HCC status based on the GALAD score was constructed to obtain the ROC curve for GALAD. The AUC with 95% CI was calculated to assess the performance of the GALAD score in the diagnosis of HCC. During the analysis, the AUC was recalculated such that controls with high AFP, AFP-L3%, or DCP (*n* = 14) beyond generally accepted high-specificity cut-offs for the three biomarkers (i.e., AFP > 400 ng/mL; AFP-L3% > 35%; and/or DCP > 2.85 ng/mL) were either imputed as cases (sensitivity analysis #1) or removed from the analysis altogether (sensitivity analysis #2). The Youden index was used to determine the optimal cut-off value for diagnosis of HCC after which sensitivities and specificities were calculated. A refit of the GALAD model was performed using logistic regression including all five individual factors [gender, age, AFP-L3%, log(AFP), and log(DCP)] to obtain new GALAD score coefficients optimized for the Ghanaian population. Subsequently, model selection was done using a backward selection process. A *P* value threshold of 0.1 was used for this process to identify a final model. Three-fold cross validation was performed to compare the AUC of the three different GALAD score models (original, full model for Ghana, and model selection). Statistical analysis was carried out using SAS version 9.4.

### Ethical approval

The study was approved by the ethical review committees of the Cape Coast Teaching Hospital (CCTHERC/RS/EC/2016/3), Korle Bu Teaching Hospital (STC/IRB/0001/2017), and Komfo-Anokye Teaching Hospital (CHRPE/AP/305/18). Written informed consent was obtained from study participants before collecting clinical information and blood samples. Studies were conducted in accordance with recognized ethical guidelines as per the Declaration of Helsinki.

### Data availability

The data generated in this study are available upon request from the corresponding author.

## Results

### Clinical characteristics of Ghanaian cirrhosis and HCC cohort

The study included 171 patients comprising 78 patients with HCC (cases) and 93 patients with cirrhosis (controls). Table 1 summarizes the key demographic and clinical characteristics of the study cohort. The median age of patients with HCC in this cohort was 45 years. There was a higher proportion of males (70.8%) in the study cohort, with no significant difference in gender between the HCC and cirrhosis groups (*P* = 0.54). HBV infection was present in 69.2% of patients with HCC, whereas HCV was present in 5.1% of patients with HCC. There was no significant difference in HBV or HCV status between HCC cases and cirrhosis controls. The median AFP (5010.2 vs. 3.4), L3% (28.3 vs. 0.3), and DCP (507.6 vs. 0.2) levels were all significantly higher among HCC cases than the controls (*P* < 0.01). The median GALAD score was also higher among patients with HCC (8.0 vs. −4.1, *P* < 0.01).

**Table 1:** Clinical characteristics of Ghanaian cirrhosis and HCC cohort

	Cirrhosis (*N* = 93)	HCC (*N* = 78)	Total (*N* = 171)	*P* value
Gender				0.54^a^
Female	29 (31.2%)	21 (26.9%)	50 (29.2%)	
Male	64 (68.8%)	57 (73.1%)	121 (70.8%)	
Age (years)				0.36^b^
*N*	89	68	157	
Mean (SD)	45.1 (11.8)	46.9 (11.4)	45.9 (11.6)	
Median (IQR)	44 (35–53)	45 (38.5–56)	44.0 (37–55)	
HBV status				0.17^a^
Negative	38 (40.9%)	24 (30.8%)	62 (36.3%)	
Positive	55 (59.1%)	54 (69.2%)	109 (63.7%)	
HCV status				0.53^a^
Negative	90 (96.8%)	74 (94.9%)	164 (95.9%)	
Positive	3 (3.2%)	4 (5.1%)	7 (4.1%)	
AFP ng/mL				<0.01^b^
*N*	93	78	171	
Median	3.4	5010.2	9.9	
Q1, Q3	2.0, 11.5	13.0, 87779.8	2.4, 5548.0	
AFP-L3%				<0.01^b^
*N*	91	77	168	
Median	0.3	28.3	8.9	
Q1, Q3	0.3, 11.1	8.0, 66.1	0.3, 32.9	
DCP ng/mL				<0.01^b^
*N*	93	78	171	
Median	0.2	507.6	0.5	
Q1, Q3	0.1, 0.4	0.8, 2654.7	0.2, 512.2	
GALAD score				<0.01^b^
*N*	87	67	154	
Median	−4.1	8.0	−2.1	
Q1, Q3	−5.5, −2.0	2.2, 13.6	−4.5, 7.5	
AST U/L				<0.01^b^
*N*	73	58	131	
Median	47.3	97.2	64.0	
Q1, Q3	30.9, 88.5	56.4, 190.0	35.1, 120.0	
ALT U/L				<0.01^b^
*N*	74	58	132	
Median	33.8	48.5	37.8	
Q1, Q3	22.0, 51.1	31.0, 97.0	25.7, 66.6	
Platelet × 10^9^/L				<0.01^b^
*N*	67	48	115	
Median	105.0	186.5	141.0	
Q1, Q3	67.0, 169.0	138.5, 269.5	89.0, 207.0	
INR				0.21^b^
*N*	37	35	72	
Median	1.3	1.5	1.4	
Q1, Q3	1.1, 1.7	1.2, 1.8	1.1, 1.8	
Albumin g/L				0.05^b^
*N*	72	54	126	
Median	31.9	35.9	34.0	
Q1, Q3	24.1, 40.2	28.0, 41.0	25.2, 41.0	
Total bilirubin μmol/L				0.79^b^
*N*	72	55	127	
Median	22.2	26.2	24.0	
Q1, Q3	15.2, 40.3	15.0, 43.0	15.0, 42.0	
Direct bilirubin μmol/L				0.88^b^
*N*	66	52	118	
Median	9.9	11.1	10.0	
Q1, Q3	6.2, 23.2	5.7, 27.0	5.9, 24.0	
Creatinine μmol/L				<0.01^b^
*N*	58	45	103	
Median	89.5	67.7	78.1	
Q1, Q3	71.0, 116.0	56.0, 95.0	62.0, 105.1	
Child–Pugh score				0.90^a^
A	12 (41.4%)	12 (36.4%)	24 (38.7%)	
B	10 (34.5%)	13 (39.4%)	23 (37.1%)	
C	7 (24.1%)	8 (24.2%)	15 (24.2%)	
Missing	64	45	109	
BCLC stage				^a^
A	N/A	4 (16.0%)	4 (16.0%)	
B	N/A	6 (24.0%)	6 (24.0%)	
C	N/A	14 (56.0%)	14 (56.0%)	
D	N/A	1 (4.0%)	1 (4.0%)	
Missing	N/A	53	53	

Abbreviations: ALT, alanine aminotransferase; AST, aspartate aminotransferase; INR, international normalized ratio.

a χ^2^.

b Kruskal–Wallis.

### Performance of the GALAD score

**Figure 1:**
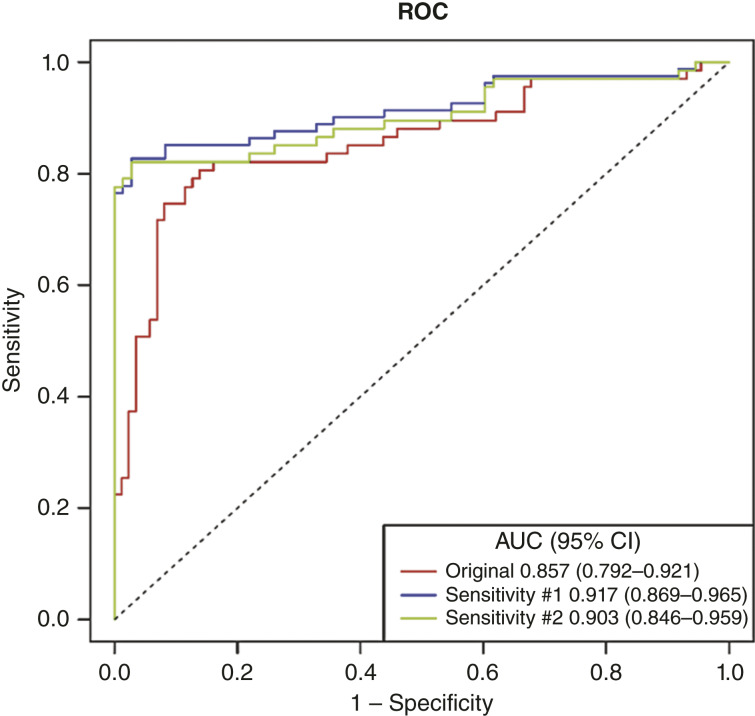
ROC of GALAD scores for the detection of HCC. *X* axis: specificity; *Y* axis: sensitivity. Sensitivity #1; controls with high AFP (>400 ng/mL), high AFP-L3% (>35%), or high DCP (>2.85 ng/mL) are imputed as cases (*N* = 14). Sensitivity #2; controls with high AFP (>400 ng/mL), high AFP-L3% (>35%), or high DCP.

The AUC of the GALAD score in the detection of HCC was 0.86 (95% CI, 0.79–0.92; Fig. 1). When controls with a high AFP, AFP-L3%, or DCP (*n* = 14) were imputed as cases (sensitivity population #1), the AUC was 0.92 (95% CI, 0.87–0.96). When these controls were removed from the analysis (sensitivity population #2), the AUC was 0.90 (95% CI, 0.85–0.96). Using the Youden index optimal cut-off value of −0.31, the GALAD score sensitivity was 0.81 and the specificity was 0.86 (Table 2).

**Table 2:** Optimal cut-off of the GALAD score with sensitivity and specificity

Cut-off	Sensitivity	Specificity
−0.31	0.81	0.86

### Stratification by hepatitis B status

There was a variation in the performance of the GALAD score depending on the HBV status, with better performance in HBV-positive persons. The AUC (95% CI) was 0.87 (0.80–0.95) and 0.81 (0.67–0.94) in HBV-positive and HBV-negative patients, respectively.

### Model refit in the Ghanaian cohort

To determine which variables in the originally published GALAD model were most predictive of HCC in our cohort, a refit of the logistic regression model was performed to obtain new coefficients (Table 3). Following a backward selection process (Table 4), the most predictive variables identified were AFP-L3% and log(DCP) (*P* < 0.01). The AUC values of the new GALAD score models using new coefficients were 0.87 and 0.89 for the full and selected models, respectively (Table 5).

**Table 3:** Refit of the logistic regression model

Variable	Original GALAD	New estimate	S.E.	*P* value
Intercept	−10.08	−2.35	1.04	0.024
Gender	1.67	0.022	0.4955	0.97
Age	0.09	0.017	0.0191	0.36
log(AFP)	2.34	0.21	0.1721	0.23
AFP-L3%	0.04	0.022	0.0015	0.048
log(DCP)	1.33	0.66	0.1587	<0.001

**Table 4:** Refit of the logistic regression model using the backward selection process

Variable	Estimate	S.E.	*P* value^a^
Intercept	−1.32	0.28	<0.001
Gender	Removed at step 1 (*P* = 0.97)		
Age	Removed at step 2 (*P* = 0.36)		
AFP-L3%	0.029	0.0098	0.0028
log(AFP)	Removed at step 3 (*P* = 0.19)		
log(DCP)	0.75	0.15	<0.001

a Backward model selection with *P* value threshold = 0.1.

**Table 5:** Comparison of the AUC of the three different GALAD score models

Model	AUC
Original	0.86
New (full model)	0.88
New (model selection)	0.89

## Discussion

In this study, the GALAD score demonstrated high accuracy (AUC 0.86) in the diagnosis of HCC in an African cohort. The median levels of AFP, AFP-L3, and DCP were found to be higher among patients with HCC than those with cirrhosis. The GALAD score achieved excellent performance (AUC 0.92) when patients with an AFP of >400 ng/mL were imputed as cases in our analysis. Furthermore AFP-L3% and DCP were significant variables in the GALAD score predicting HCC, whereas age and gender were not. Refitting the GALAD model further enhanced the model performance for HCC detection.

The GALAD score has been validated in studies across Europe, Asia, and the Americas, where results have demonstrated good diagnostic accuracy of the model (15–17). A 2023 systematic review reported a pooled sensitivity of 82%, specificity of 89%, and AUC of 0.92 across 15 studies (20). In a cohort of patients with HCC in Germany where the predominant cause of HCC was alcohol, the AUC of the GALAD score was 0.9, with a sensitivity of 81.2% and specificity of 85.5% at a cut-off value of −0.65 (23). Among patients in the United States with HCC predominantly due to HCV and alcohol, the sensitivity and specificity of the GALAD score were 71.4% and 78.5%, respectively, with an AUC of 0.84 at a cut-off value of −0.63 (24). In China, where similar to Ghana, HBV is the main risk factor for HCC, the GALAD score demonstrated an AUC of 0.84, similar to our study, with sensitivity of 81% and specificity of 72.8% at a cut-off of −0.63 (25). In this study, the optimal cut-off value with 81% sensitivity and 86% specificity was −0.31.

The GALAD model incorporates AFP, AFP-L3%, and DCP, as well as gender and age. Individually, these biomarkers show variable performance in HCC diagnosis. AFP levels are elevated in hepatic inflammation, liver regeneration, and HCC development. However, the biomarker has been found to be less specific than AFP-L3, which is produced by HCC cells (26). In an analysis of 147 studies, the performance of AFP in the diagnosis of HCC at a cut-off threshold of 20 ng/mL was found to be 60% and 84% for sensitivity and specificity, respectively. A cut-off threshold of 200 ng/mL demonstrated a sensitivity and specificity of 36% and 99%, respectively, for any-stage HCC in an analysis of 56 studies (27). AFP-L3%, which is calculated as the ratio of AFP-L3 to AFP, has a specificity of 95% in the diagnosis of HCC (20). DCP, which is also known as protein induced by vitamin K deficiency or antagonist-II (PIVKA-II), is found in the serum of patients with HCC and has been found to complement AFP in the diagnosis of HCC, with an AUC of 0.86 when used in combination with AFP in diagnosing early-stage HBV-related HCC (28).

In our study, the median levels of AFP, AFP-L3%, and DCP were higher among patients with HCC than controls, with the most significant biomarkers in the score being AFP-L3% and DCP. Age was not a significant variable in the model; this may be because HCC occurs at a younger age in sub-Saharan Africans compared with other parts of the world (4). In our study, the median age for patients with HCC was 45 years, which is much younger than other GALAD validation studies, where the median age of patients ranged between 53 and 75 years (20).

In Ghana and several other sub-Saharan African countries, there are no formal HCC surveillance programs, and early detection of HCC remains challenging (29). As demonstrated in our cohort, most patients presented with advanced BCLC stages B, C, and D (for HCC cases) and higher Child–Pugh score classes B and C (for HCC cases and cirrhosis controls). This late stage of presentation is consistent with studies conducted in Africa (4, 5) and emphasizes the need for improved surveillance of patients in the region who are at risk of HCC, in order to diagnose disease at an earlier stage when curative therapy is an option. Regional guidelines are being developed for Africa, and in a survey of practitioners, roughly 60% of practitioners from Africa reported the use of abdominal ultrasound and AFP as their surveillance modality of choice for HCC in accordance with international societal guidelines (9, 30), whereas 8% utilize AFP alone and 21% use abdominal ultrasonography (US) alone (31). Newer diagnostic scores and biomarkers are yet to be incorporated into guidelines. Consequently, AFP and US remain the main recommended surveillance modalities. Due to limited access to and operator dependency of abdominal US especially in low-resource settings where the number of well-trained sonographers is inadequate (11, 32), there is a need to identify diagnostic tests that are simple to use, easy to interpret, and have superior performance as alternate or complementary surveillance modalities. Consequently, further large-scale multi-country studies of the performance of the GALAD score either alone or in combination with US are warranted in Africa and may inform region-specific guidelines for HCC. GALAD assays are available outside Africa, and if the evidence of its utility is demonstrated in African countries, one may then bring forth the case to industry partners to make the assay available on the continent with one of the highest incidences of HCC, at a reasonable price.

This study had several limitations. The diagnosis of HCC and cirrhosis was based on clinical history, physical examination, laboratory, ultrasound, and where available CT scan findings. No FibroScan was performed due to lack of availability. As patients had to pay for investigations out of pocket, not all investigations could be undertaken by all patients on account of financial constraints. Therefore, abdominal CT scans or AFP levels could not be obtained for some patients, and this could have led to misclassification of some cases as controls. Furthermore, information on antiviral treatment was not collected for patients. As antiviral therapy is known to suppress baseline AFP levels and improve the performance of AFP in surveillance testing, this could have influenced the results of the analysis. Additionally, due to limited staging information, the performance of the GALAD score in early stage (BCLC 0 or A) could not be assessed in this study and will be pursued in a follow-up study. Further studies in a larger cohort of patients from Africa, including patients with earlier stages of HCC (BCLC 0/A), are needed to improve the statistical power, better validate GALAD, determine the role of age in the GALAD model in sub-Saharan African patients, and make its use more generalizable to the African population.

### Conclusion

The GALAD model demonstrated good performance in diagnosing HCC in Ghanaian patients with predominantly HBV-associated HCC. This score or its relevant modifications have the potential to aid in improving HCC surveillance efforts in low-resource settings in sub-Saharan Africa.
